# Supplementary material for: The Potential Regulation of A-to-I RNA Editing on Genes in Parkinson’s Disease
Source: Genes (Basel). 2023 Apr 15;14(4):919. doi: 10.3390/genes14040919 (PMC10137963; doi:10.3390/genes14040919)
Supplement: Supplementary file 1 [file genes-14-00919-s001.zip › SupplemtaryFigures.pdf]

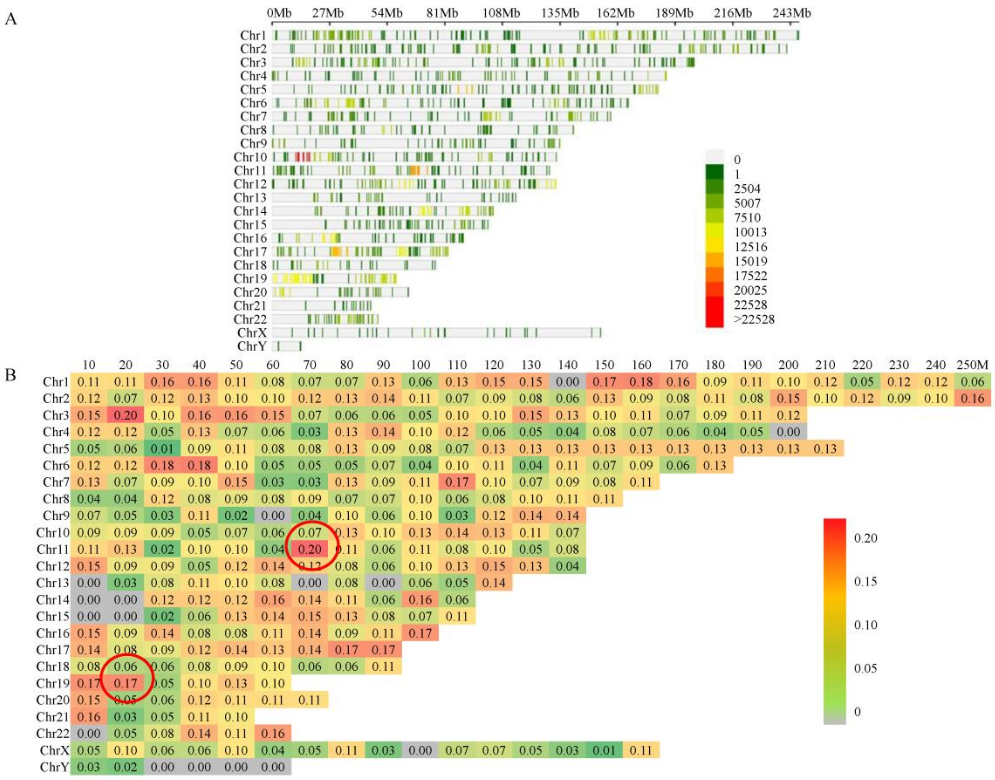

Figure S1. The density of RNA editing events and genes on chromosomes. (A) The density of gene-associated RNA editing events on chromosomes. The color bar shows the number of gene-associated editing events in a 10M bps (base pairs) region. (B) The ratio of the number of editing-associated genes and the number of all the genes across each genomic region (10M bps) of chromosomes. It describes the density of editing-associated genes, removing background noise.

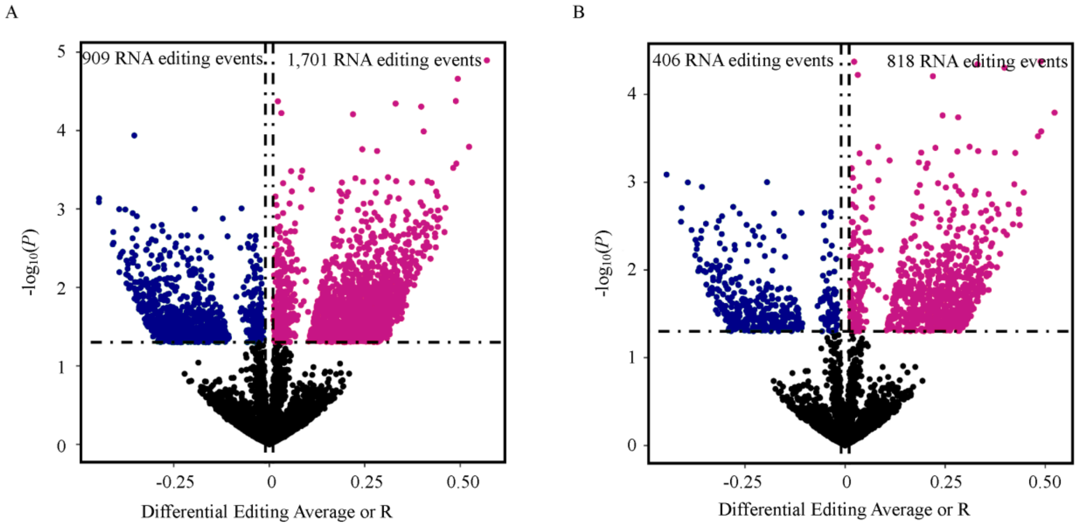

Figure S2. The frequency analysis of gene-associated RNA editing events. (A) Among the 9,897 RNA editing events, there are 2,610 events differentially edited in PD or in more severe PD samples ($P < 0.05$). (B) Among the 4,623 RNA editing events associated with the expressions of 821 PD-related genes, there are 1,224 events differentially edited in PD or in more severe PD samples ($P < 0.05$).

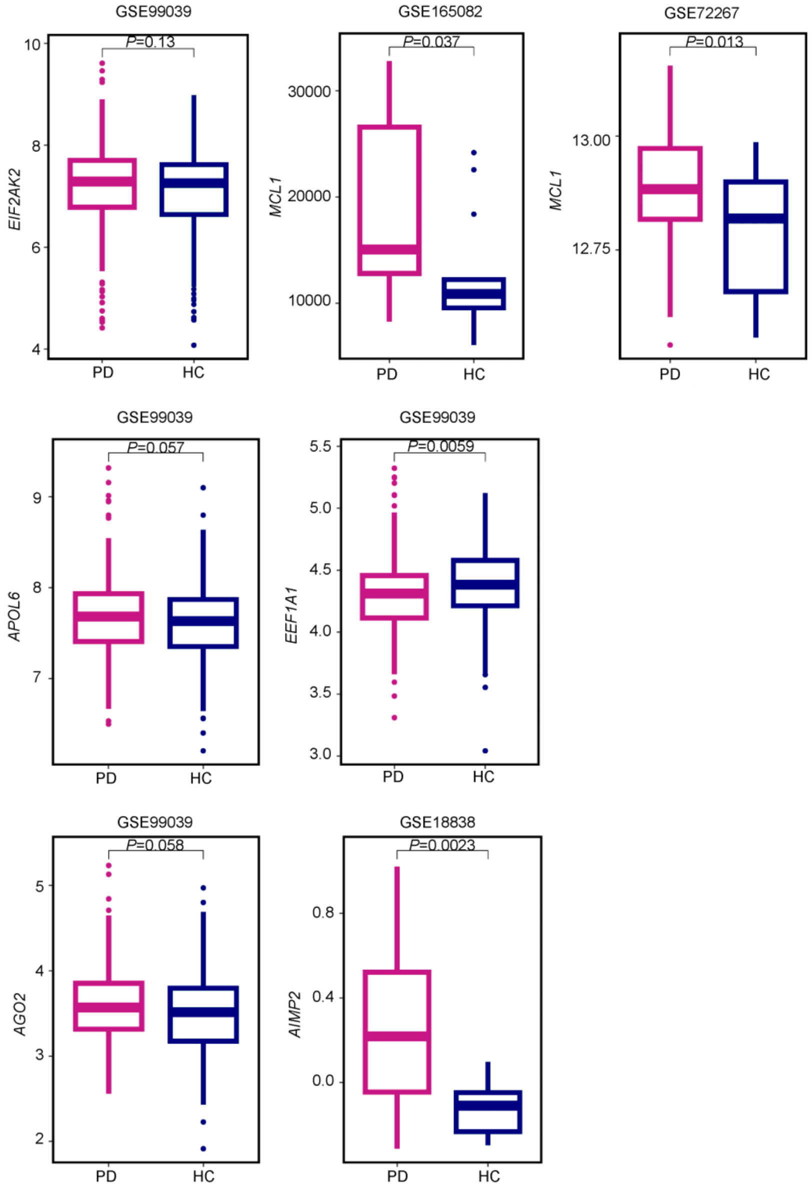

Figure S3. The comparisons of the six genes introduced in this study between PD samples and healthy controls (HC) in four GEO datasets. The comparison results of (A) *EIF2AK2*, (B-C) *MCL1*, (D) *APOL6*, (E) *EEF1A1*, (F) *AGO2*, and (G) *AIMP2* between PD samples and healthy controls. For GSE165082 dataset, it shows the read counts obtained from RNA sequencing samples. For the datasets of GSE72267, GSE99039 and GSE18838, it shows the normalized expression values calculated by the pipeline of Robust Multi-array Average (RMA).

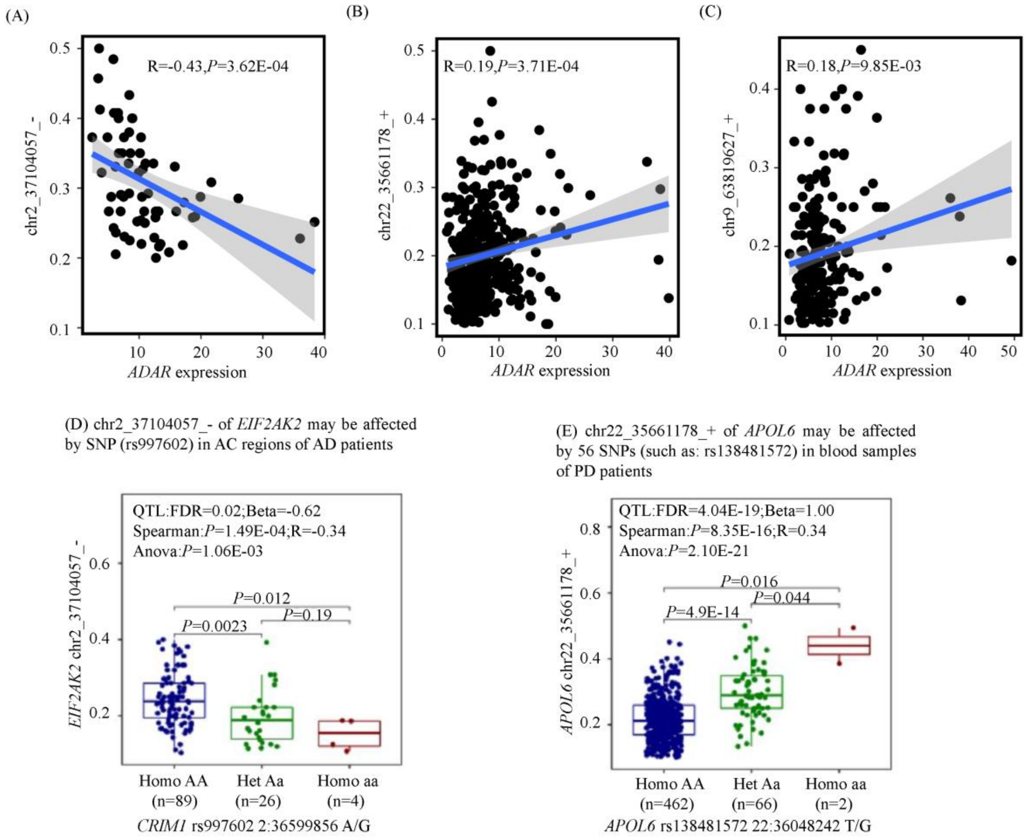

Figure S4. The potential up-regulators of A-to-I RNA editing biomarkers. (A-C) The correlations of *ADAR* enzyme with the three RNA editing events. (D-E) The effects of genetic variants on the two RNA editing events.

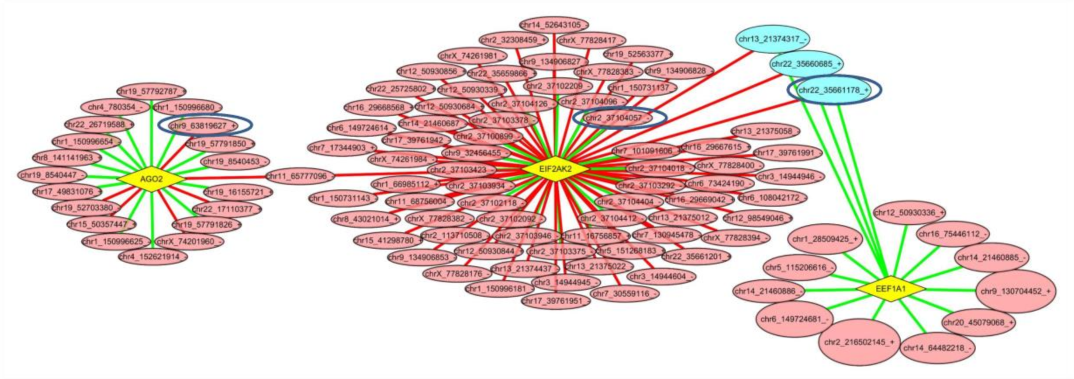

Figure S5. The effects of multiple RNA editing events on the three genes. These RNA editing events were all predicted to disturb miRNA regulations. The red and green lines present the positive and negative effects of RNA editing events on genes. The light blue circles represent the RNA editing events displaying possibly opposite roles on the two genes. The three RNA editing events introduced in this study are highlighted by navy circles.

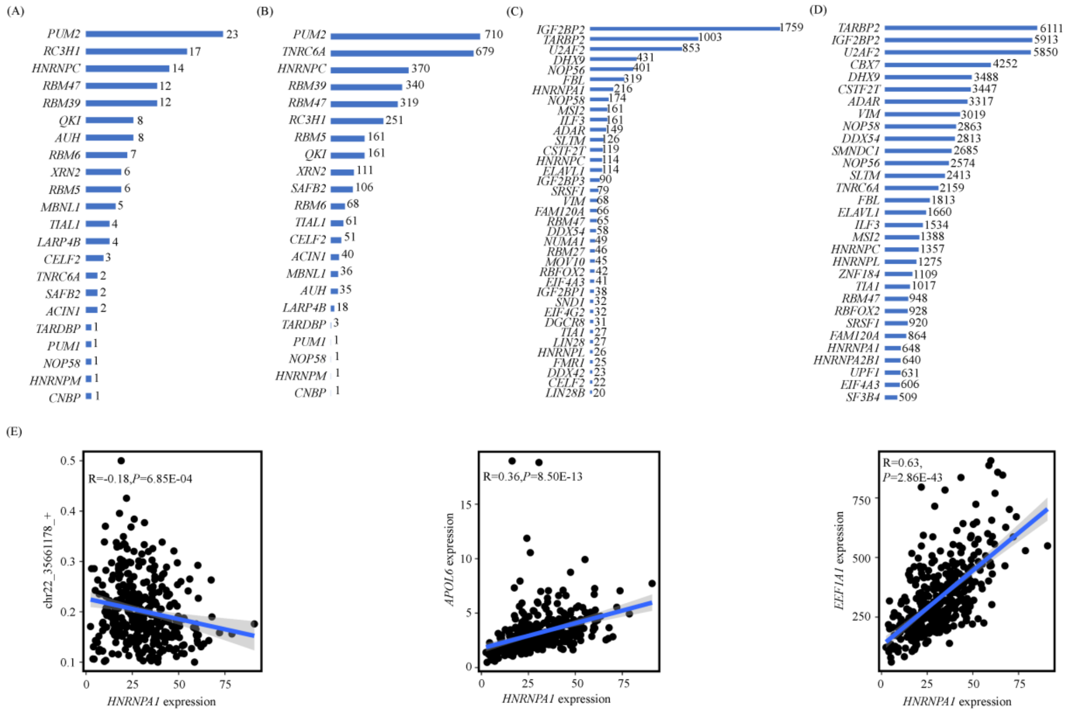

Figure S6. The potential involvement of RNA editing events in the regulations of RNA-binding proteins (RBP) on genes. (A) The number of RNA editing events in RBPs. (B) The number of genes associated with the RNA editing events in RBPs. (C) The number of RNA editing events in RBP targets. (D) The number of genes associated with the RNA editing events in RBP targets. (E) The correlations of an RNA-binding protein, *HNRNPA1*, with the editing event (Chr22:35661178), *APOL6*, and *EEF1A1*. This RNA editing event is located in the binding region of *HNRNPA1*, which may interfere in the regulations of this RNA-binding protein on the two genes.
